# The Active Hospital pilot: A qualitative study exploring the implementation of a Trust-wide Sport and Exercise Medicine-led physical activity intervention

**DOI:** 10.1371/journal.pone.0257802

**Published:** 2021-09-24

**Authors:** Anna Myers, Helen Quirk, Anna Lowe, Helen Crank, David Broom, Natasha Jones, Hamish Reid, Chris Speers, Robert Copeland

**Affiliations:** 1 Advanced Wellbeing Research Centre, Sheffield Hallam University, Sheffield, United Kingdom; 2 Academy of Sport and Physical Activity, Sheffield Hallam University, Sheffield, United Kingdom; 3 School of Health and Related Research, University of Sheffield, Sheffield, United Kingdom; 4 National Centre for Sport and Exercise Medicine-Sheffield, Sheffield Hallam University, Sheffield, United Kingdom; 5 Centre for Sport, Exercise and Life Sciences, Coventry University, Coventry, United Kingdom; 6 Oxford University Hospital NHS Foundation Trust, Oxford, United Kingdom; 7 Centre for Sport and Orthopaedic Medicine, Hamilton, Bermuda; University of Montreal, CANADA

## Abstract

**Background:**

In 2017 Public Health England and Sport England commissioned a Consultant-led Sport and Exercise Medicine (SEM) pilot to test the feasibility and acceptability of embedding physical activity interventions in secondary care clinical pathways. The aim of this paper is to report qualitative findings exploring the experience of healthcare professionals (HCPs) and patients involved in the Active Hospital pilot.

**Methods:**

Qualitative data was collected by semi-structured interviews with Active Hospital pilot SEM Consultants, and staff and patients involved in three clinical pathways. Interviews with SEM Consultants explored the experience of developing and implementing the pilot. Interviews with staff and patients explored the experience of delivering and receiving Active Hospital interventions. Data were analysed thematically.

**Results:**

Interviews identified the importance of the Active Hospital pilot being Consultant-led for the following reasons; i) having trusting relationships with decision makers, ii) having sufficient influence to effect change, iii) identifying champions within the system, and iv) being adaptable to change and ensuring the programme fits within the wider strategic frameworks. HCPs emphasised the importance of the Active Hospital interventions fitting easily within existing work practices, the need for staff training and to tailor interventions for individual patient needs. The Active Hospital pilot was well received by patients, however a lack of dedicated resource and capacity to deliver the intervention was highlighted as a challenge by both patients and HCPs.

**Conclusion:**

The SEM Consultants’ ability to navigate the political climate of a large National Health Service (NHS) Trust with competing agendas and limited resource was valuable. The interventions were well received and a valued addition to usual clinical care. However, implementation and ongoing delivery of the pilot encountered challenges including lack of capacity within the system and delays with recruiting to the delivery teams in each pathway.

## Background

Physical inactivity is the fourth leading cause of death worldwide [1, 2]. It accounts for approximately 6% of all deaths globally and is directly responsible for a huge burden of non-communicable disease (NCD) [3]. Most NCDs can be ameliorated through being more physically active [4]. Inactive individuals are more likely to develop chronic illness, which is a well-recognised cause of activity reduction [3]. This means people with chronic health conditions are amongst the least active members of society despite having the most to gain from even small increases in PA [5].

Healthcare has been highlighted as one of the ’7 best investments’ for the promotion of physical activity (PA) to reduce the burden of NCDs and promote population health [6]. This provides a unique opportunity for healthcare professionals (HCPs), who are valued as trusted and credible source of PA information, to encourage their patients to become more physically active [7]. Sedentary hospital in-patients are at risk of deconditioning [8] resulting in reduced muscle strength and mass (sarcopenia), decline in functional ability leading to frailty [9], longer hospital stays, and deterioration in quality of life, particularly in older adults [10, 11]. Furthermore, the decline in PA and increase in sedentary behaviour observed during hospitalisation can be maintained post-discharge even when patients have recovered to full physical function and are medically stable [9, 12]. Patients exposed to mobilisation (such as walking) during hospitalisation show significant improvement in physical function, shorter hospital stays, fewer complications, such as pulmonary embolism and are at no greater risk of falls [13].

Progress has been made in developing PA pathways in disease-specific silos in outpatient settings (e.g. cardiac rehabilitation, see Dalal, Doherty [14]), in primary care settings (e.g. exercise referral schemes, see Pavey, Anokye [15]) and in the prevention of hospital related deconditioning in specific populations (e.g. older adults, see Scheerman, Raaijmakers [16]. However, little ground has been made with embedding PA within routine inpatient care at a system level [17]. The opportunity to prevent hospital-associated deconditioning is being missed. This is a key omission in healthcare’s contribution to improving population PA since leadership from a specialist service has been found to be fundamental to a system wide change in culture in the National Health Service (NHS) [18].

The translation of evidence into practice is challenging. Traditional approaches have taken a rational-linear approach where knowledge is created by experts and passed on to another set of experts to be implemented [19, 20]. Yet this approach ignores the context of the wider system within which interventions are implemented [21]. Acknowledging and embracing the complexity of systems such as the NHS can help to achieve successful implementation of evidence-based interventions [22].

### The Active Hospital pilot

In 2017, as part of the ‘Moving Healthcare Professionals Programme’ [23], Public Health England (PHE) and Sport England commissioned a Consultant-led Sport and Exercise Medicine (SEM) pilot to test the feasibility and acceptability of embedding PA interventions in secondary care; the Active Hospital pilot [24]. Historically, SEM in the NHS has focused on musculoskeletal clinical pathways, but with training in exercise medicine, population health, musculoskeletal medicine and multidisciplinary team work, SEM physicians are uniquely positioned to support a transition toward empowering patients to lead active lifestyles, particularly when working collaboratively with a multidisciplinary team [25, 26].

**Fig 1:**
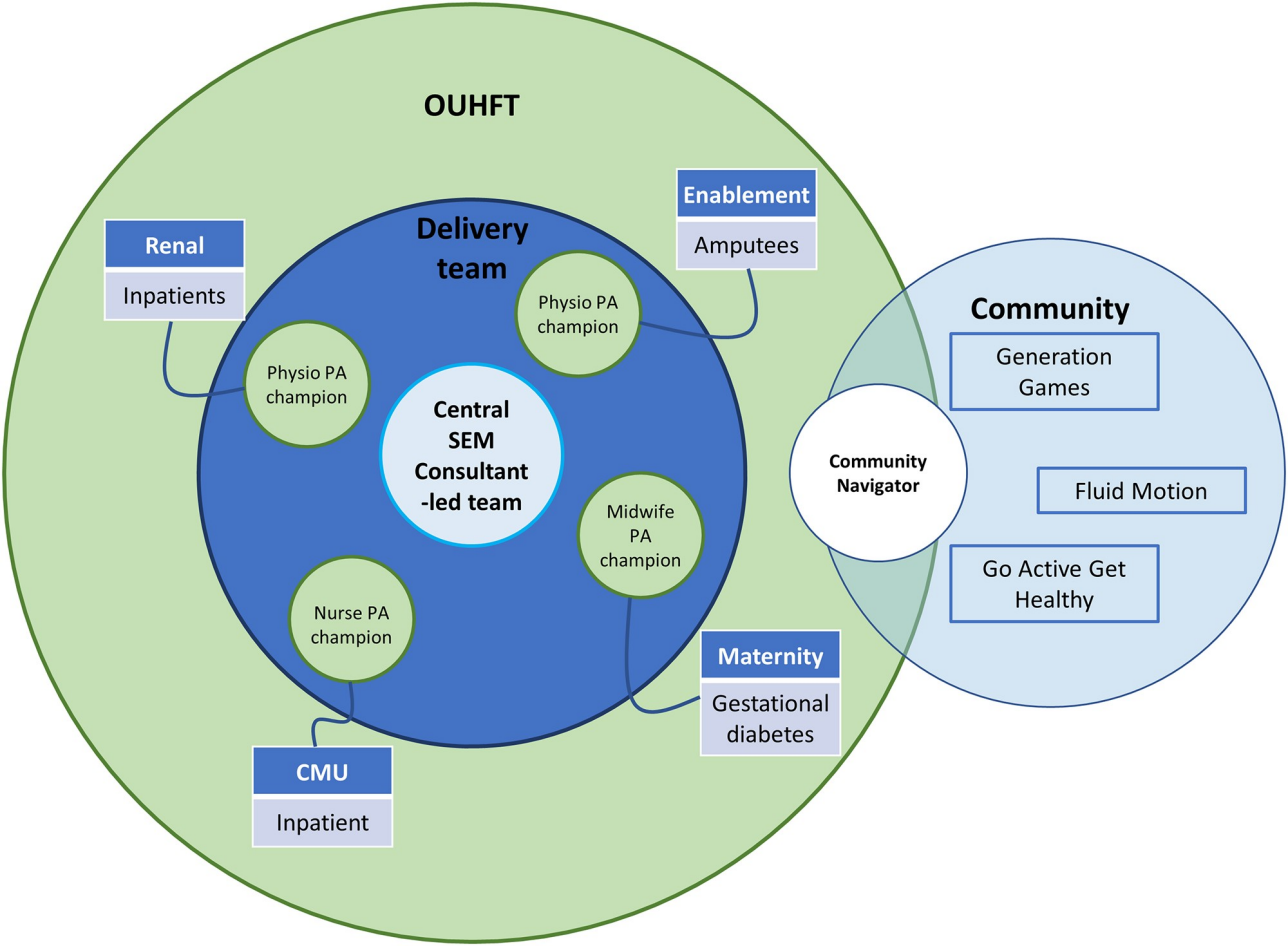
Schematic representation of the Active Hospital pilot SEM Consultant-led model. Qualitative evaluation was conducted in three of the four clinical pathways (Enablement, Renal and CMU). (OUHFT, Oxford University Hospital Foundation Trust; PA, physical activity; SEM, Sport and Exercise Medicine;; CMU, complex medical unit).

The aim of the Active Hospital pilot was to embed a SEM Consultant-led PA service within Oxford University Hospital Foundation Trust (OUHFT). This was tested across four clinical pathways where PA had not previously been targeted as a treatment intervention and patients have multiple LTCs and significant symptomatology; Maternity (high risk pregnancy), Enablement (limb deficiency), Renal and Complex Medical Unit (CMU) (see Fig 1). The Active Hospital pilot was a multi-component complex intervention; each pathway had a different intervention protocol to suit the varying physical and cultural environments, each with the aim of increasing in-hospital and post-discharge PA levels.

Development and planning for the Active Hospital pilot began in December 2017 and the implementation phase followed in January 2018 and continued until March 2019. The Active Hospital team at OUHFT mapped the interventions for each pathway against behaviour change techniques using the COM-B (’Capability’, ’Opportunity’, ’Motivation’ and ’Behaviour’) model [27]. Table 1 provides details of the intervention components for each pathway. Further information can be found in the evaluation report [28].

**Table 1:** Overview of the aims and intervention components for each of the four clinical pathways involved in the Active Hospital pilot at OUHFT.

Pathway	Aims	Intervention components
**Maternity**	i) Increase PA behaviour of pregnant women attending the Silver Star and gestational diabetes management (GDM) services.	Demonstrate types of activities that are safe in pregnancy (i.e. provision of Chief Medical Officers’ PA guidelines for pregnant women) during clinical appointments and through educational resources (i.e. posters in waiting areas).
		Goal setting and motivational interviewing (including identifying motivators and barriers to PA) offered to patients who are flagged as high risk.
		Provide sensible advice regarding how PA can be built into daily life, signpost and promote opportunities locally.
		Increasing knowledge and awareness by educating patients on the benefit of PA during their pregnancy.
		Use of positive imagery to motivate women to increase their PA, for example, display case studies in posters and This girl can posters in Maternity waiting areas.
	ii) Increasing the frequency of PA brief advice interventions for pregnant women given by staff.	A PA calculator was integrated into the Maternity booking form on the electronic patient record. This triggered on screen prompts to ask about PA vital signs and then patients were given a leaflet on PA.
		Key members of staff trained by the central OUHFT Active Hospital pilot team (see Table 2) to deliver brief PA advice to pregnant women using motivational interviewing and goal setting during consultations.
		Education on the local PA resources, common barriers to PA and how to address them. Observation session to encourage staff to maintain change in practice. Feedback from patients regarding delivery of motivational interviewing session or brief intervention. Support Service for staff with email advice and drop-in sessions for challenging cases.
		System designed to support HCPs who have limited time and need the resources to hand to signpost patients on for more support. This consisted of the provision of PA infographic in clinical rooms.
		Explore opportunities for a reward system for those staff attending training sessions and delivering PA advice (e.g. PA champion of the month and ‘I have had my PA training’ badge.
**Enablement**	i) Encourage PA by providing patients access to a PA class and a peer support group for medical amputees.	A group-based PA class to support the physiotherapy rehabilitation programme that is open to medical amputees and their partners. The class incorporates exercises that can be taught in a gym-based setting and transferred to a home-based setting. Classes consist of demonstration of simple exercises in a safe environment with the opportunity for feedback and assistance.
		Classes could accommodate up to five patients. Each class was led by the PA champion and a Physiotherapist. A second physiotherapist was available if required. The classes were delivered in a circuit format and involved rotating around different activities.
		Patient facing PA resources (e.g. posters) displaying positive imagery of PA in gyms and clinic rooms and messages on the Enablement ward such as on-screen prompts and posters to prompt staff to engage with discussions regarding PA.
		Identify motivators and barriers to PA amongst patients and their partners before, during and after the classes and provide personalised exercises and goals.
		Integrate exercises involving readily available items to facilitate PA and supply patients with the equipment required to complete the class at home where necessary.
		Educational messages regarding the benefits/importance of PA delivered during classes.
		Attendance monitoring and participants encouraged to keep a PA diary.
		*N*.*B*. *The intention was to development of peer support group with education sessions*. *However*, *no formal peer mentors were recruited or trained within the lifecycle of this study*. *At the time of concluding this study*, *two patients had been approached to become peer mentors and the Enablement team were awaiting a response*.
	ii) Impart staff with the knowledge regarding the importance of PA and empower them to integrate PA advise into their routine practice.	Key members of staff in the Enablement pathway trained by the central OUHFT Active Hospital pilot team (see Table 2) to deliver brief PA advice using motivational interviewing and goal setting.
		Develop educational materials regarding PA with Enablement staff and distribute to patients. Staff in the Oxford Centre for Enablement received PA training delivered by SEM registrar and Consultant. They also had motivational interviewing training through active conversations class. Staff were involved in the development of the Amputee Moving Medicine module and development of the patient information resources. Patients were given an information leaflet about the benefits of PA for people with amputation (this was developed alongside the amputee Moving Medicine module). The exercise class programme was available as a patient information leaflet and a patient workbook was used to support motivational interviewing conversations.
		Develop on screen prompts for staff to discuss PA.
		Distribute positive feedback regarding PA discussions from patients to staff. Informally–verbal feedback, compliments, and examples of patients with positive outcomes from PA interventions were shared during team meetings, planning meetings and governance meetings. Formally–positive feedback was shared in presentations about the pathway delivered by SEM Registrar and in poster presentations for conferences.
**Renal**	i) Develop an activity permissive environment (active ward) and a social support network to foster peer-to-peer support to increase the PA of in-patients on the Renal ward.	Development of a PA permissive culture by encouraging twice daily walking in and around the ward with other patients, family and where available staff.
		Walking was encouraged through the provision of signed routes, seating in long corridors for rest stops and the removal of barriers such as locked doors.
		Educate patients on the benefits of being physically active both on their general health and active symptoms. Extending this knowledge and permission to families and friends.
		Identify and manage patients’ psychological barriers to increasing PA on the ward.
		Minimise times when patients are expected to be confined to their rooms.
		Posters and patient information on the benefits of PA as well as how and where to be active on the ward.
		*N*.*B*. *Social network to foster peer-to-peer support*. *This element of the pathway proved difficult to implement and no peer mentors were recruited within the study lifecycle because no patients were willing to be peer mentors*.
	ii) Impart staff with the knowledge regarding the importance of PA and empower them to integrate PA advise into their routine practice.	Key members of staff in the Renal pathway (i.e. transplant nurses and ward staff) trained by the central OUHFT Active Hospital pilot team (see Table 2) to deliver brief PA advice using motivational interviewing and goal setting.
		Support the development of a ward and clinical environment that supports promotion of PA.
		Develop tools and ward systems to support frequent conversations about PA between HCPs and patients.
		Develop prompts to encourage professional to asks the PA vital sign and provide ongoing brief PA advice and consistent messaging to patients.
**Complex Medical Unit (CMU)**	i) implement a patient centred PA intervention to increase the amount of PA patients do during their stay on the ward.	PA champion encouraged patients who had a stable Early Warning Score and were not on an end of life pathway to be more physically active using motivational interviewing.
		A patient workbook was used during motivational interviews to give consistent structure to the conversation, help patients set their ambitions and plan how to achieve their goals.
		Bed-based, chair-based and standing exercise programme booklets were available for patients depending on their physical ability.
		Development and implementation of the ‘I CAN’ tool that documents each patients’ physical capability so ward staff are aware of what the patient can do. The intention of the ‘I CAN’ tool is to overcome risk aversion by giving permission for patient to move more rather than traditional bed rest. Further information about the ‘I CAN’ tool can be found here https://movingmedicine.ac.uk/active-hospitals/find-resources/
	ii) Impart staff with the knowledge regarding the importance of PA and empower them to integrate PA advise into their routine practice.	Key members of staff in the CMU pathway trained by the central OUHFT Active Hospital pilot team (see Table 2) to deliver brief PA advice using motivational interviewing and goal setting.
		All conversations regarding PA were documented in the patient’s Electronic Patient Record.

Table 2 describes the roles within the Active Hospital team at OUHFT. The delivery model was chosen to ensure that rapid learning from each pathway could be shared by staff already working within that pathway with knowledge of the patient journey and opportunities for change.

**Table 2:** The OUHFT Active Hospital pilot delivery team structure.

Active Hospital pilot role	Job role	Role description
**Central team**—leads	SEM consultant (n = 2)	The SEM pilot was led by two SEM Consultants. A senior SEM Consultant and a locum SEM Consultant (the person undertaking this role left after 12 months in post and was subsequently replaced in January 2019). The SEM Consultants led the development and implementation of the pilot, including the COM-B mapping process and governance structure for the interventions. They were the main point of contact between the central team and the delivery teams within each pathway. The SEM Consultants provided bespoke PA training based on the Moving Medicine (https://movingmedicine.ac.uk/) evidence review to the PA champions who then cascaded the training to the delivery team within their pathway. The training focused on the health benefits of PA and behaviour change techniques–namely motivational interviewing.
**Central team**—support	SEM Registrar (n = 4)	The SEM registrars led the delivery of each clinical pathway. The SEM Registrars and a band 6 Physiotherapist led the COM-B mapping process and established the governance structure for the interventions across each pathway.
	Physiotherapist (n = 1)	The Physiotherapist also provided one-to-one training in motivational interviewing to PA champions which was followed-up with observed consultations and feedback.
**Delivery team**—Clinical pathway leads	Consultant (n = 3)	A local lead from each pathway was identified to improve and agree pathway interventions and ensure service wide buy in.
	Ward Sister (n = 1)	
**Delivery team**—Physical activity champions	Maternity–Midwife (n = 1)	The role of the PA champions was to: i) deliver the interventions, ii) provide the central team with information and learning from individual pathways, iii) cascade training to ward staff, encourage staff to promote PA and embed culture change, and iv) undertake governance activities including audit, quality improvement and data collection to ensure evidence-based medicine, education and training.
	Enablement–Physiotherapist (n = 1)	
	Renal–Physiotherapist (n = 1)	
	Complex Medical Unit–Nurse (n = 1)	
**Community support**	Community navigator (n = 1)	A Community Navigator and Community Navigation map was available to help patients and staff identify community-based exercise teams and classes during the discharge process to ensure integrated care.

In line with guidance outlined in the ‘Standard evaluation framework for physical activity interventions’ [29], PHE and Sport England commissioned the National Centre for Sport and Exercise Medicine—Sheffield (NCSEM), in partnership with Sheffield Hallam University (SHU), to conduct an independent evaluation of the Active Hospital pilot. Members of the evaluation team met with the OUHFT Active Hospital pilot leads in December 2017 to identify the active components of the intervention(s), establish core evaluation outcomes, determine an agreed position for the evaluation and identify the most appropriate data collection methodology. This paper explores the experience of HCPs and patients involved in the Active Hospital pilot across three clinical pathways. The aim of the study was to explore how the pilot was implemented, how it was experienced by SEM Consultants, HCPs and patients, what worked well and what challenges were encountered.

## Methods

The overall approach to understanding the experience of implementing the Active Hospital Pilot was guided by a case-study approach [30] which utilised multiple methods to explore broad areas of a) overall experience, b) what worked well, and c) what were the challenges across programme elements. We considered the entire Active Hospitals pilot as one case study, as opposed to each individual pathway being a separate case study. Case Studies are particularly suited to exploring how services are implemented within and through specific organisational processes and contexts and often comprise synthesis of multiple forms of data [30]. Here we report the qualitative data that informed the overall evaluation, which is reported elsewhere [28]. We undertook semi-structured interviews with Active Hospital pilot SEM Consultants, staff and patients involved in three of the four clinical pathways (Enablement, Renal and CMU). The qualitative interviews were substantiated with on-site observations at OUHFT by the evaluation team at three time-points during the Active Hospital pilot (July and October 2018 and February 2019). A quantitative approach was taken to the evaluation of the Maternity pathway based on the design of the PA intervention therefore there was no qualitative data to report for this pathway. All study procedures and documentation were reviewed and approved by the National Research Ethics Service Committee Nottingham 2 (ref. 18/EM/0145). Findings from the qualitative interviews and on-site observations were then mapped against the three overarching, strategic principles of the Successful Healthcare Improvement From Translating Evidence in complex systems (SHIFT-Evidence) framework [22]. SHIFT-Evidence reflects the nature of work, and breadth of effort, required to translate evidence into complex systems. The SHIFT-Evidence framework suggests that to make and sustain improvements from evidence translation in healthcare, it is necessary to act scientifically and pragmatically whilst embracing the complexity of the setting in which change takes place and engaging and empowering those responsible for and affected by the change.

### Recruitment

Participants were recruited via the SEM Consultants at OUHFT between July 2018 and February 2019 using a purposeful sampling procedure. Potential interviewees were identified pragmatically, based on their involvement in the Active Hospital pilot and availability for interview. All potential interviewees received a participant information sheet and had the opportunity to contact the research team to ask any questions before providing written informed consent to participate. Signed consent forms were received either electronically or on hard copy and stored securely by the research team. A mutually convenient time and interview format (face-to-face or telephone) was arranged.

### Participants

Seventeen people were invited to interview and 13 took part. Two patients declined the invite to an interview and two patients did not respond to the invite. Reasons for refusing an interview were not sought or recorded. Participants were SEM Consultants responsible for the development and implementation of the Active Hospital pilot (n = 3; face-to-face); HCPs providing care in the Renal pathway (n = 2; face-to-face) and CMU pathway (n = 3; 2 telephone and 1 face-to-face); and patients receiving treatment in the Renal pathway (n = 1; telephone) and Enablement pathway (n = 4; 2 telephone and 2 face-to-face) at OUHFT. The SEM Consultants agreed for their roles to be disclosed in this manuscript and acknowledge it might be possible to identify them from the information provided. HCPs within each pathway who were interviewed, or demographic details of patients have not been disclosed to protect their anonymity. Interview duration ranged from 12 to 69 min and the average duration was 39 min.

### Data collection

Four researchers (two male, two female) conducted the interviews (RC, HC, DB and HQ). All researchers were experienced qualitative researchers with combined expertise in PA, psychology, exercise science, behaviour change and programme evaluation. Three researchers (HC, DB, HQ) did not have any prior relationship with interviewees. RC was known to the SEM Consultants he interviewed, but none of the researchers had any role in the implementation or delivery of the Active Hospital pilot. All interviewees were informed that the researchers were responsible for conducting the evaluation of the Active Hospital pilot.

Interviews took place in-person (n = 8) and via the telephone (n = 5). Researchers checked each participant’s understanding and consent before starting the interview. Only the researcher (interviewer) and interviewee were present during the interview. To ensure consistency across interviews, semi-structured interview guides were used by all researchers conducting the interviews (see S1 File for interview guides). The interview guides were developed pragmatically by the research team to address the aims of the evaluation. The interview guides did not undergo pilot testing due to time constraints, but the research team did go through a series of iterations and revisions in the development of them. The SEM Consultants completed two interviews each, given their key leadership role in the development and implementation of the pilot. The first interview with the SEM Consultants was in the early implementation phase of the Active Hospital pilot (July 2018) and the second at the end of the pilot (February 2019). One interview was conducted with each HCP and each patient towards the end of the pilot (between December 2018 and March 2019).

### Data analysis

Data analysis was an iterative process. All interviews were audio-recorded using a digital recorder and transcribed verbatim by an independent transcription company. Transcripts were not returned to participants for comments or correction due to time constraints. Data was analysed thematically, following an approach akin to the ‘codebook’ thematic analysis method described by Braun and Clarke [31], which used a coding framework for developing and documenting the analysis. This approach was appropriate to ensure that the data answered the pre-determined research questions and satisfied the purpose of the Active Hospital evaluation. Four researchers (HQ, RC, HC and DB) were involved in the analysis of data. Interview transcripts were read and re-read several times by the researchers. In addition, the recordings were listened to ensuring the accuracy of the transcription and to aid familiarisation and interpretation. Transcripts were coded primarily on a priori concepts guided by central questions and concepts. Concepts explored in the framework included a) overall experience, b) what worked well, and c) what were the challenges. Codes were then organised into themes that best described the perspective being explored. The four researchers involved in the analysis of data carefully explored the data collected from each perspective and a further two researchers (AM and AL) provided critical questioning of the analysis. Participants did not provide feedback on the data analysis. Quotes are used to exemplify key aspects of the interviewees’ experiences and opinions and to help clarify links between data, interpretation and conclusions.

## Results

The experiences of the SEM Consultants responsible for implementing the Active Hospital pilot are presented and discussed followed by results from the three clinical pathways; Enablement, Renal and CMU. Qualitative data was not captured in the Maternity pathway and therefore we were unable to explore the experience of HCPs and patients in that pathway.

### Experience of the SEM Consultants in developing and delivering the Active Hospital pilot

#### The importance of leadership that is trusted and embedded within the system

Implementation of the Active Hospital pilot was complex and challenging. SEM Consultants highlighted the importance of strong leadership across the system which was facilitated by long-standing relationships the senior SEM Consultant had with staff across the Trust. The senior SEM Consultant took overall responsibility for the project with service delivery overseen by the locum. The senior SEM Consultant made introductions between the locum and key staff across the Trust and ensured that the locum was able to navigate their way around the hospital–culturally and politically, as well as structurally. The senior Consultant’s ‘intuitive’ knowledge of the Trust gained through over 10 years experience was a significant advantage when implementing the pilot.

*"I think it would be very difficult for a new Consultant to come into a Trust and immediately set up a new service like this. I think you need to know people both on the clinical and the managerial side, or it makes it an awful lot easier if you do. I’m not saying it wouldn’t be possible, I just think it would take longer."*

*(SEM Consultant 2)*

The culture change observed at OUHFT did not happen overnight, but was driven by influential individuals with a clear vision for change:

*"These things generally get driven by influential people with a strong desire for change and so when we’re looking to scale this model, we need to have things that are going to enable those people to start making changes."*

*(SEM Consultant 1)*

The SEM Consultants believed that effective conversations that positively influenced engagement with the Active Hospital pilot occurred at the top level of the hospital’s hierarchical system. For example, because the senior SEM Consultant had a background in general medicine, they were able to have conversations with Consultant leads in other services in a more direct manner than perhaps a non-medical HCP might be able to. The model of having a Consultant at the top is also consistent with other services in the hospital system. Moreover, any scepticism about non-medical interventions like PA held by clinicians and managers was believed to be easier to overcome due to the existing trust between established Consultants at the top level. The experiences described by the SEM Consultants suggest that it would perhaps be more difficult if these conversations needed to happen across different hierarchical levels and with staff who are new to the Trust.

*"Where there’s a senior clinician, a Consultant who is engaged in driving forward some of these initiatives in a department that makes a huge difference in terms of the impact there and that really impacts upon the speed at which you can make stuff happen."*

*(SEM Consultant 1*)

Having the SEM Consultants embedded at the top level of the system was believed to facilitate conversations with commissioners within the Trust. The SEM Consultants identified that the offer to commissioners needed to include the contribution that SEM will make to the day-to-day activity of the Trust and not just on the basis of a culture change programme to promote PA, even where the rationale was that it might save money in the future.

*"I think from a Trust [perspective] they need to have the easy quick wins*. *They need to know that you can see 50 patients a week and you can bring in the money that way…we’ll do that for three days a week and then for the other two days I’m going to do this PA intervention*, *I think that’s easier"*

*(SEM Consultant 2)*

#### Create the team from a coalition of the willing to drive change

Data from the interviews highlighted the importance of developing a ‘network of PA champions’ within departments and services so that lines of communication between the delivery teams in each pathway and the central team could easily be maintained.

*"The PA champion network works so well because you have someone that’s in that service and you’re talking to them regularly*, *and if there’s problems in an area you can go and chat to the people in that service a bit higher up the food chain or something like that to see what the barriers are and explore things*.*"*

*(SEM Consultant 1)*

One of the roles of the SEM Consultants was to identify opportunities for collaboration and establish a coalition of the willing. To do this, the SEM Consultants needed to have the skills, influence and mandate to ‘join the dots’ of practice that was already occurring in the Trust. This had the effect of creating momentum across departments that was beyond what was being implemented directly through this pilot.

*"The more you find people are doing stuff*, *like one of the vascular surgeons has set up milestones for walking down the ward and things like that*. *So there are people doing stuff like this and this is where having a central service to support it and share ideas and drive change is so valuable really*, *because all these small things that people are already working on and doing brilliantly that work in different environments have just never been coordinated*.*"*

*(SEM Consultant 1)*

To achieve this, the SEM Consultants described spending time having meetings with a range of people across the Trust to promote engagement and talk about what they can do, what they can offer, and how PA and SEM skills can fit into existing services.

The SEM Consultants identified that the PA champions, as points of contact, need to be embedded in pathways. The PA champions needed to be given the freedom and mandate to lead change locally and needed to be identified early in the process:

*"Having someone already in the department to be able to be there and initiate*, *so we’ve got a therapy assistant working with amputees who was working part-time as a therapy assistant and part-time in research and he was allowed to come out of his research time to deliver this*.*"*

*(SEM Consultant 1)*

#### The importance of governance structures to ensure quality service improvements and sustainability

The SEM Consultants were also required to develop new leaders of culture change within different services so that once their direct support was withdrawn, the services were sustained. It was not deemed sufficient to simply appoint the team leads and kick-start the intervention without a longer-term planning and auditing process. One way to achieve this was through establishing policies and structures that aligned to making the new behaviours sustainable. These policies then needed to be bolstered with good governance and follow-up processes.

*“support them with a governance framework and the quality improvements*, *the strategic vision*, *but actually withdraw our direct input*.*”*

*(SEM Consultant 2)*

The Consultant-led team operated within a governance framework which mandated regular audit and Quality Improvement Projects (QIP), patient and public involvement, staff education and training adherence to deliver evidence-based interventions.

*"if you set it up but leave them and you never go back to them*, *it might sustain for another year*. *So*, *what you need to do is set things up*, *then leave them*, *but keep going back and reminding them*, *do some audits*, *do the patient feedback*, *do the health professional feedback*, *do the 360 appraisals*, *all those things as part of a really robust governance structure and that will sustain it but that all takes work and it takes leadership*.*"*

*(SEM Consultant 2)*

The system leadership, central co-ordination functions provided by the SEM Consultants and governance framework were also essential in the sustainability of the interventions. Throughout the Active Hospital pilot they acted as voices for change and provided a platform upon which to continue to build. Had the support of this core team been removed, evidence here suggests that the pilot would have potentially collapsed.

*"Everyone’s getting engaged and they’ve made a big effort at it*, *but then the reality is that it’s hard to maintain*, *because to sustain that change takes a lot more than just a good idea and an intervention*. *They have to be embedded in the day to day practice of that specific department"*

*(SEM Consultant 1)*

#### A flexible approach and a willingness to fail

The Active Hospital pilot had clearly articulated plans for how and where PA was to be embedded within the Trust. Over time, it became clear that changes to the implementation process needed to be made, either because clinical pathways were simply inappropriate for the proposed interventions and/or because there were existing activities that could be harnessed to drive forward a change in culture, but were not part of the original scope. The SEM Consultants needed to be sensitive to where these new opportunities existed whilst at the same time being adaptable to change and looking for opportunities across the system to intervene.

*"You just shoehorn things in*, *I don’t think it’s the best way of doing things but because of the reality of the timelines we’ve just been jumping on any opportunity we can and making it happen*.*"*

*(SEM Consultant 1)*

The SEM Consultants also needed to be able to recognise that the contexts differed significantly between departments and this was facilitated by a baseline understanding of what was currently being delivered in the Trust, where it was being delivered and by whom.

*"We might be working on general medical wards one ward to the next ward and the routines will be different*, *the staffing patterns will be different*, *when they do the drug rounds will be different*.*"*

*(SEM Consultant 1)*

These qualities of flexibility also applied to the development of resources. The implementation of activities as a one-size-fits all approach would not work due to the varying contexts within the Trust services.

*"It has to be very individualised within [departments]*, *so you work from the core elements*, *but then the implementation has to be tailored*, *so there’s that difficult balance between making common resources to support everybody*, *but having flexibility that they can be adapted into whatever the demands of the specific service are*.*"*

*(SEM Consultant 1)*

In addition, several practical aspects to the set-up of the Active Hospital pilot were highlighted that could have enhanced the approach to implementation. These included, but were not limited to, the early employment of staff on the programme, finance systems being established at the outset, and system prompts setup through the Electronic Patient Record. This is the ideal scenario of course and this needs to be tempered with the realities of implementing change within a resource stretched Trust with multiple competing agendas. This is where the experience, connections and trusted relationships of the senior SEM Consultant were perhaps most valuable.

### Findings from the three clinical pathways

#### Enablement pathway

The four participants (patients) who were interviewed had participated in an Enablement PA class (see Table 1 for details). Patients described enjoying taking part in the class and praised the staff for providing support that was empathetic, motivational and patient-focused. Patients valued HCPs who had the knowledge and the interpersonal skills to create patient rapport and support patient motivation.

*"And they’ve all been very good to me [HCPs]*. *The whole journey from when I lost my legs has been good*.*"*

*(Enablement patient 4)*

*"He’s [HCP] allowed me to talk and he’s very understanding*, *empathetic*. *He’s a top bloke really"*

*(Enablement patient 2)*

Patients perceived that services were under-resourced and that increasing staffing capacity would be needed to enhance the capacity of future PA interventions within the Enablement pathway.

*"my answer when it comes to the NHS*: *there’s not enough of them [HCPs] and there isn’t enough money*, *it’s as simple as that*. *The [HCPs] we have don’t need any kind of improvement at all*. *We just need more of them"*

*(Enablement patient 2)*

The group-based format of the PA class was deemed motivational and it provided the opportunity for tailored patient support whilst also accommodating several patients at once. The PA class did not involve any formal peer mentors but peer support was experienced, with patients valuing the opportunity to share their experiences through forming connections and having informal chats with other class attendees who shared a mutual understanding of their experience as amputees.

*"It’s not just one aspect of it*. *It’s the whole group working together and the encouragement of everybody that’s there*, *not just the physios and the doctor*. *It’s everybody else*, *as patients*, *helping each other out*. *And I think it’s good we can talk about what happened to us because it is massively life changing"*

*(Enablement patient 2)*

A peer-to-peer support mentor role was planned as part of the PA intervention, but no patients were recruited or trained as intended within the evaluation lifecycle. At the time of concluding this study, two patients had been approached to become peer mentors and the Enablement team were awaiting a response. The intention to support others who have been through something similar was strong among the patients interviewed. However, not all patients interviewed were supportive of a formal peer support mentor role. For some patients being asked to formally engage in conversations with others that might be perceived as personal or uncomfortable was not appealing. Previous history of other conditions such as mental health conditions, personal preferences, logistical issues (i.e. physical ability to get to venue) also meant some patients did not wish to engage in peer-to-peer support programmes. This is perhaps underlined by the fact that no formal peer mentors were recruited or trained as was intended. Mentoring is clearly a complex process and it is likely that it will require the patient mentor to have certain competencies to deliver successful outcomes. Setting, processes, communication skills and mentor characteristics therefore need careful consideration. Moreover, it cannot be assumed that the peer-to-peer support model will suit everyone even where these competencies are met.

#### Renal pathway

One patient and two HCPs who had been involved in the Renal pathway ’active ward’ (see Table 1) were interviewed. A peer mentoring scheme was planned for the Renal ward however, it became apparent that it would not be feasible for several reasons including short stay admissions, a lack of social space and the need for patient isolation to reduce the risk of infection. The pathway evolved to include a daily walking rounds, individualised motivational support, goal setting and posters (see Table 1 for more details). The HCPs interviewed highlighted the benefits of having an active ward as well as the need for sustainability. They felt that capitalising on the ’teachable moment’ early after surgery might act as a catalyst for positive behaviour change. Both HCPs were overwhelmingly positive about the importance of using motivational interviewing with patients and the led walks were deemed to be of value.

*"Over the last few months we’ve been seeing the kidney transplants mainly for motivational interviewing and just getting them talking about PA… So the motivational interviewing sessions have probably been the biggest thing*. *So I sit down with patients and ask them about PA*. *How they were before their surgery and if doing a bit more since their surgery is something they want to do"*.

*(Renal HCP 1)*

However, the medical equipment Renal patients often require on the ward was a barrier to the mobilisation of patients and took time to get patients to the walk meeting point. Furthermore, HCPs perceived a clear difference in the level of ability, fitness and willingness to undertake PA in the Renal patients.

*"And also with varying levels of ability among the transplant patients*, *some people are really active*, *some people not so much*. *And I think putting them all in a walking group together was quite difficult… The patients that haven’t been so appropriate tend to be the ones that are fitter already and don’t really need our support with that"*.

*(Renal HCP 1)*

*"There was also the difficulty of people being at very different levels*. *From somebody that’s very active before they came to hospital to somebody that can only really stand for a few minutes before they came to hospital*.*"*

*(Renal HCP 2)*

HCPs believed that all staff members in the multidisciplinary team have the responsibility to promote PA to patients and buy-in from staff across disciplines and levels is important. A staffing crisis on the Renal ward was said to make implementation difficult, but there was a clear desire to keep the promotion of PA high on the agenda. Staff were keen to maintain visibility of the programme to ensure its sustainability, which meant posters were important.

*"I think it’s been slightly difficult for us in particularly because we’re really short staffed*. *We’re missing a couple of band 5s…so ideally we’d be getting patients up into the gym and getting them doing exercise as soon as possible*. *But sometimes we’re a little bit delayed with that which can’t be helped because a nurse needs to be with them at all times"*.

*(Renal HCP 1)*

*"On the Renal ward they were having a real crisis of staffing when we first started"*.

*(Renal HCP 2)*

Whilst only one patient agreed to be interviewed, they were complimentary about their experience on the ward as well as the PA education and opportunities they received. They felt that PA should be a part of every patient’s treatment on the Renal ward, but it needs to be tailored to individual patient needs and ability. The patient would be happy to be a mentor or mentee in a formal peer-to-peer support mentor programme, but they felt that training would be needed to become a peer mentor.

*"I’m not clever enough to know what [exercises] that would be*. *What would be a good idea is that the part of the pack you get when you come out is*, *while you are in there some information is given about the local centres"*.

*(Renal HCP 3)*

#### Complex Medical Unit (CMU) pathway

Three HCPs who were involved in the CMU pathway were interviewed. The aim of the PA intervention on the CMU was to change the culture of the ward such that PA and mobilisation were encouraged to all patients (wherever possible) and by all staff. HCPs perceived a clear need for this intervention on their ward because mobilisation of patients on the CMU ward was a priority, to prevent pressure areas and infections. It was regarded a positive move towards a shift in culture for both ward staff and patients.

*"Most people that I’ve come across have been really pleased about doing it*. *They’ve enjoyed it*. *Sometimes they’ve been surprised about what they’ve managed and it’s generally been well received by patients"*

*(CMU HCP 3)*

*"it’s the knock-on effect…it only takes one in the bay and someone else will say tomorrow*, *well he’s walking*, *Alfie’s walking to the toilet*, *I’d like to do that*.*"*

*(CMU HCP 2)*

The implementation of the Active Hospital pilot in the CMU pathway included the use of exercise booklets and the ‘I CAN’ tool (which documents a patient’s physical capability) with patients on the ward. The staff identified training, changes to practice and having dedicated nurse PA champions as facilitators of implementation.

*"what’s made it easier*, *obviously having people having some dedicated time for this*, *and having some dedicated champions on the ward that are nurse based"*

*(CMU HCP 3)*

It was apparent that physiotherapists on the ward were already well accustomed to mobilising patients, whereas this was not always a priority for nurses. The ‘I CAN’ tool and the exercise booklet were praised for their ease of use and for facilitating communication between staff, especially staff across different shifts. The staff interviewed were hopeful that the ‘I CAN’ tool would encourage patients to keep mobile to prevent deconditioning and maintain functional capacity.

*"It’s found to be helpful*, *especially for communication between members of the team when they come to work"*

*(CMU HCP 1)*

*"[the exercise booklets] can be distributed by any member of staff*, *they’re fairly self-explanatory"*

*(CMU HCP 3)*

HCPs emphasised the importance of the intervention fitting easily within the system and existing work practices. An example of this was taking an existing pressure sore prevention and treatment tool (SSKIN: Surface, Skin infection, Keep your patient moving, Incontinence/moisture, Nutrition/hydration) and placing more emphasis on the ‘**K**eep your patient moving’ element of this tool, rather than introducing it as a new procedure. Anecdotal evidence from the staff suggested that the exercises to keep patients moving were well received by those patients who experienced them.

*"I have seen a nurse doing exercises with a patient*. *The patient looked very involved and interested and taking it seriously as something good for his health*, *and as a positive really*.*"*

*(CMU HCP 1)*

Staff noted that that the exercises were not suitable for all patients, for example those with palliative care needs, suggesting that an individualised and case-by-case approach to implementation is needed.

## Discussion

This study explored the experience of developing, delivering and being a recipient of the Active Hospital pilot from the perspective of the SEM Consultant leads, HCPs and patients. We discuss the findings from qualitative interviews that are mapped against the three overarching, strategic principles of the Successful Healthcare Improvement From Translating Evidence in complex systems (SHIFT-Evidence) framework [22].

### Act scientifically and pragmatically

Knowledge of existing evidence needs to be combined with knowledge of the unique initial conditions of a system, and interventions need to adapt as the complex system responds and learning emerges about unpredictable effects. The senior SEM Consultant reported having an ‘intuitive’ knowledge of the Trust having worked in the context for over 10 years. Being embedded within the higher levels of the system meant that the Consultant understood how the system behaved [32]. Creating a network of embedded PA champions with intricate knowledge their respective systems meant the central team could utilise local expertise when designing and implementing the interventions. The PA champions acted as a feedback mechanism to the central teams enabling them to intervene and adapt the intervention if required.

### Embrace complexity

Evidence-based interventions only work if related practices and processes of care within the complex system are functional, and evidence-translation efforts need to identify and address any problems with usual care, recognising that this typically includes a range of interdependent parts of the system. Care pathways are complex interventions within complex systems [33]. The Active Hospital pilot could be described as a small system (or microsystem), as could each of the pilot pathways, which sits within the Trust. Each of these small systems is nested within a series of larger, interconnected systems within the site, which itself is nested within the Trust, which is in turn nested within the broader place-based health economy. The SEM Consultants’ role in connecting these different systems and negotiating with different change agents across the system was important for success. Trusting and enduring relationships with other Consultants were deemed important—particularly at the outset of the pilot when making the case for change and engaging staff in each of the pathways.

Being able to navigate the political climate of a large Trust is challenging and change programmes such as the Active Hospital pilot are potentially at risk of getting overshadowed by larger agendas without strong senior leadership. This is supported by findings from the implementation of staff health and wellbeing services in NHS secondary care, which also showed that the support from senior leaders within the Trust is imperative for success [34]. Future research and evaluation should seek to explore whether programmes like Active Hospitals can be delivered by trusted members of staff that might not be at the top of the hierarchy. Where these long-standing relationships do not already exist, future programmes might need to lengthen any ‘set-up’ phase to factor in for relationship building and development of trust.

### Engage and empower

Evidence translation and system navigation requires commitment and insights from staff and patients with experience of the local system, and changes need to align with their motivations and concerns.

Whilst the Consultant-led approach was important, the SEM Consultants also emphasised the importance of identifying PA champions within the system to drive change at the local level, describing a coalition of the willing as essential. The PA champions on the ground were deemed to be important for ensuring the sustainability of the pilot, meaning the Consultants were not the ‘lynch pin’ for success. Success also depended on being adaptable to change, working to the strengths of the system and choosing to implement interventions in areas demonstrating most enthusiasm and engagement. A mapping exercise helped to identify these paths of least resistance and the COM-B approach to intervention mapping ensured the interventions were underpinned by behaviour change theory and helped engender some consistency across the system. A robust clinical governance framework was also implemented that mandated regular audit and QIP, risk management, patient and public involvement, and staff education and training. These governance measures were key to improving the quality of care within the pilot pathways and ensuring the sustainability of the interventions. The SEM Consultants suggested that they deal with clinical governance on a regular basis and so this is perhaps an important reason why a Consultant role is well suited to leading this type of intervention. Across each pathway, HCPs emphasised the importance of the intervention fitting easily within the system and existing work practices, the need for staff training and to tailor interventions for individual needs.

The SHIFT-Evidence framework identifies that providing headroom, resources, training, and support is critical to make and sustain change [22]. The Active Hospital pilot created these conditions within the Trust, it enabled focused work with shared aims and objectives and gave key actors within the system the ’headspace’ to implement evidence-based PA interventions. The extent to which the project has ongoing legacy is critical in understanding true impact and the next phase of this work will see other NHS Trusts implement an Active Hospital approach.

### Strengths and limitations

The main strengths of this evaluation relate to its methodological rigour. Using an external evaluation team meant that the evaluation was independent of OUHFT (developed and implemented the pilot) and Sport England/PHE (provided funding). The evaluation team identified several methodological challenges, which should be considered when interpreting the findings. Recruitment was challenging and only small numbers of HCPs and patients were interviewed. Recruitment was purposeful, pragmatic and led by the SEM Consultants, introducing potential bias to the findings. The small sample means it is possible that a range of different viewpoints were not captured from the wider multidisciplinary teams and patients involved in the pathways. Additionally, qualitative data was not captured in one of the four Active Hospital pilot pathways (Maternity) and therefore we were unable to report the experience of HCPs and patients in that pathway and patient experience was not captured in the CMU pathway. Furthermore, only one patient was recruited in the renal pathway. The results should therefore be interpreted with caution. It is also possible that self-selection bias resulted in a sample that may be skewed towards reporting outcomes from patients and HCPs who are engaged and supportive of the pilot. When considering the potential for the Active Hospital pilot to bring about change, it is important to be realistic about the time it may take for change to happen. Embedding the programme into the Trust is likely to require a change in culture and therefore any changes brought about by the pilot might not be expected to manifest as observable change within the lifespan of this evaluation.

### Key considerations for practice

Several considerations were identified for future interventions that aim to embed PA within secondary care. These are points for consideration rather than direct recommendations.

1. A consultant-led approach facilitates reaching agreements, achieving a mandate for action and deciding how interventions can be implemented. Where a Consultant-led (or equivalent) approach is not feasible or appropriate, programme delivers should factor in the time needed for the leadership team to develop trusting relationships with those working on the ground. A more modest approach focussing on fewer clinical pathways might be appropriate to provide the time and space required to focus on forging trusted relationships and becoming embedded within the system.
2. Undertaking a mapping exercise at the outset of the pilot to explore the physical and social environment of the hospital, who is working where, what can be fitted in where, what is being done well in the hospital, where things can be augmented and where the key partners are, would be a sensible first step in implementation of future Active Hospital interventions.
3. Thinking broadly about who the stakeholders are and engaging them early to help design the intervention and how it will be delivered will increase the likelihood of the service being fit for purpose and sustainable. The project is likely to benefit from wider knowledge of, and multiple perspectives on the system. A potential limitation was the top-down approach taken to the Active Hospital pilot. The success of the pilot required buy-in from not only those leading the project but the staff delivering care on the ground. Some transfer of ownership/responsibility from the SEM Consultants to the rest of the workforce may be required to ensure sustainability of the pilot long-term.
4. Identifying key supporters or project champions on the ground may drive engagement, ownership and change. Ideally these project advocates would be from a range of professions and at every level within in the organisation. Change agents and leaders within the system may not be limited to clinical staff.
5. There should be strategic alignment with the wider priorities of the organisation and where possible with the wider health economy. This will help to make the case for change and to engage senior leadership.
6. Building in a sustainability plan from the outset will mean that any changes are meaningfully embedded within the system and will be less likely to ’fizzle out’ when the project ends or when key individuals move on.
7. Being open and reflective about failures will facilitate learning capabilities. Embedding processes which help the team to review, reflect and iterate will be key to the learning process. Flexibility and the ability to adapt quickly to change are essential.

### Conclusion

The Active Hospital pilot represented a complex system intervention delivered across multiple clinical pathways within a single NHS Trust. Data from HCPs and patients involved in the pilot indicate it was well received and a valued addition to usual clinical care, but implementation and ongoing delivery of the Active Hospital pilot was not without its challenges.

For an intervention to succeed it must be flexible and adaptable to the surrounding system and to individual patient’s needs and capability. Consistent leadership from senior clinical staff with trusting relationships and connections across the system were important enablers.

### Next steps

Following Phase one of the Moving Healthcare Professionals Programme, PHE and Sport England are undertaking a second phase of the programme launched within the Government’s prevention strategy [35]. Phase two includes exploring the transferability and feasibility of embedding the Active Hospital pilot model (developed at OUHFT) within a variety of hospital environments and within different clinical pathways.

## Supporting information

S1 File Active Hospital pilot interview guides. (DOCX)
