# Supplementary material for: The Active Hospital pilot: A qualitative study exploring the implementation of a Trust-wide Sport and Exercise Medicine-led physical activity intervention
Source: PLoS One. 2021 Sep 24;16(9):e0257802. doi: 10.1371/journal.pone.0257802 (PMC8462703; doi:10.1371/journal.pone.0257802)
Supplement: S1 File — (DOCX) [file pone.0257802.s001.docx]

**Complex Medical Unit pathway**

**Preamble**

Confirm consent and permission to record.

**Section 1 – Introductions**

- Researcher introduces themself and provides an overview of their role within the project.
- Set expectations for how long the interview will last.

**Section 2 – Opening question**

- Please can you tell me about who you are; your job role and what that entails?
- What would you consider to be priorities for patient care in your Trust?
  - Minimise patient waiting time / enhance quality of life / adherence to medication
- What factors do you think are important for a healthy lifestyle?
  - Diet / smoking / physical activity
- How do you feel about physical activity being promoted within your Trust (Oxford)?
- Whose responsibility is it to promote physical activity and exercise in general (not just health care setting)?
  - Nurse / physio / occupational therapist / community PA providers / peer mentor
- What are the opportunities to promote physical activity within your Trust?
  - What part do patients play in the promotion of physical activity?
  - What part do health care staff play?

**Section 3 – physical activity initiative and the I CAN tool**

- Are you aware of the patient centred physical activity initiative that patients have been involved with? (prompt: the I CAN tool)
- Please can you describe the initiative?
- Tell me about your experience of caring for patients who were part of the physical activity initiative?
- Has this changed how you care for other patients on the ward who are not involved with the initiative?
- Have you received any training on physical activity to encourage patient to be more active on the ward?
- How was this physical activity initiative received by patients?
- What did you think to the I CAN tool?
- What was it like caring for patients who have specific PA goals set out in the I CAN tool?
- Did it help patients to be more physically active?

**Section 4 - Process and impact**

- What do you think worked well with the physical activity initiative?
- Compared to before this initiative, what did you do differently?
- What changes did you notice in colleagues and patients on your ward?

**Section 5 - Barriers and facilitators**

- What or who made it easier for patients to be more physically active?
- What were the barriers?
- Who else do you think could / should have been involved?

**Section 6 - Sustainability and transferability**

- How sustainable is the patient centred physical activity initiative and the use of the I CAN tool?
- What would need to happen for it to continue?
- If another care pathway was to adopt this approach, what steps would they need to take?
- How transferable is the approach?
- Would you recommend the patient centred physical activity initiative and the use of the I CAN tool to other care pathways / hospitals?

**Section 7 – Close**

- Is there anything else that you have not had chance to discuss that you would like to tell me about?
- Have you got any questions for me?

**Enablement pathway**

**Preamble**

Confirm consent and permission to record.

**Section 1 – Introductions**

- Researcher introduces themself and provides an overview of their role within the project.
- Set expectations for how long the interview will last.

**Section 2 - Opening questions**

- Can you try to describe what your own journey has been as a patient in Oxford Trust?
  - Key people / key events
  - Highlights - what’s been the best thing about your journey?
  - Lowlights - what have been the challenges about your journey?

**Section 3 - Peer-to-peer support**

- How did you find out about the peer mentoring scheme?
- What interested you about the scheme?
- Please tell me about your experience of the peer mentoring scheme?
  - Who provided support?
  - How was support provided?
  - How often did you have contact with the peer support group?
- What worked well in the peer mentoring scheme?
- What influence has the peer mentoring scheme had on you?
- What, if anything, have you learnt from the peer mentoring scheme?
- What kind of person makes a good peer mentor?
  - Why are those things important?
- What do you think a peer mentor should do?
  - *Examples: regular contact (face-to-face / email / telephone / text), lead by example*
- Was the scheme what you expected?
  - If no, why?
- What do you feel were the least effective elements of the peer mentoring scheme?
  - Can you tell me why you think that?
- How could the peer mentoring scheme be improved?
- How much of an impact do you think the peer mentoring scheme has on other patients?

**Section 4 - Close**

- Is there anything else that you have not had chance to discuss that you would like to tell me about?
- Have you got any questions for me?

**Renal pathway (healthcare professional)**

**Preamble**

Confirm consent and permission to record.

**Section 1 – Introductions**

- Researcher introduces themself and provides an overview of their role within the project.
- Set expectations for how long the interview will last.

**Section 2 – Opening questions**

- Please can you tell me about who you are; your job role and what that entails?
- What would you consider to be priorities for patient care in your Trust?
  - Minimise patient waiting time / enhance quality of life / adherence to medication
- What factors do you think are important for a healthy lifestyle?
  - Diet / smoking / physical activity
- How do you feel about physical activity being promoted within your Trust (Oxford)?
- Whose responsibility is it to promote physical activity and exercise in general (not just health care setting)?
  - Nurse / physio / occupational therapist / community PA providers / peer mentor
- What are the opportunities to promote physical activity within your Trust?
  - What part do patients play in the promotion of physical activity?
  - What part do health care staff play?

**Section 3 - The Active Ward**

- What does “Active Ward” mean to you?
- What has been your involvement with the Active Ward pilot?
- Tell me about your experience of working on an Active Ward?
- If I was a new HCP and I walked on to the ward, what would I notice?
- How was it received by those involved?

**Section 4 - Process and impact**

- What do you think worked well with the Active Ward?
- Compared to before the Active Ward, what did you do differently?
- What changes did you notice in colleagues and patients on your ward?

**Section 5 - Barriers and facilitators**

- What or who made it easier to implement the Active Ward?
- Who were the key players involved in getting the Active Ward up and running?
- What were the barriers to implementing the Active Ward?
- Who else do you think could / should have been involved?

**Section 7 - Sustainability and transferability**

- How sustainable are the changes to your ward?
- What would need to happen for this to continue?
- If another ward was to adopt an Active Ward approach, what steps would they need to take?
- How transferable is the approach?
- Would you recommend this type of approach?

**Section 6 - Close**

- Is there anything else that you have not had chance to discuss that you would like to tell me about?
- Have you got any questions for me?

**Renal pathway (patient)**

**Preamble**

Confirm consent and permission to record.

**Section 1 – Introductions**

- Researcher introduces themself and provides an overview of their role within the project.
- Set expectations for how long the interview will last.

**Section 2 - Opening questions**

- Can you try to describe what your own journey has been as a renal patient in Oxford Trust?
  - Key people / key events
  - Highlights - what’s been the best thing about your journey?
  - Lowlights - what have been the challenges about your journey?

**Section 3 - The Active Ward**

- What does “Active Ward” mean to you?
- What makes an active ward different from other hospital wards? OR If I was a new patient and I walked on to the ward, what would I notice?
- What do you think about the active ward?
- What did other patients think about the Active Ward?
- Can you think of anything that might have improved the Active Ward?

**Section 4 - Impact**

- What impact did it have on you?
  - Did these changes have an impact on your condition / health / recovery?
- What differences did you notice in other patients?
- Were you involved with the peer support group?
- Do you think other wards should adopt the Active Ward approach?

**Section 5 - Close**

- Is there anything else that you have not had chance to discuss that you would like to tell me about?
- Have you got any questions for me?

**Sport and Exercise Medicine Consultants**

**Preamble**

Confirm consent and permission to record.

**Section 1 – Introductions**

- Researcher introduces themself and provides an overview of their role within the project.
- Set expectations for how long the interview will last.

**Section 2 - Opening question**

- Please can you tell me about who you are; your job role and what that entails?
- Generally speaking, what is it like to work within Oxford NHS Trust?
- What would you consider to be priorities for patient care in your Trust?
- What do you think the value of SEM is to Oxford Trust?
  - Where and how can SEM make a difference?
  - Are there challenges within the trust where SEM can make a difference?
- Whose responsibility is it to promote physical activity and exercise in general (not just health care setting)?
  - Nurse / physio / occupational therapist / community PA providers / SEMs
- What are the opportunities to promote physical activity in your Trust?
- Do you feel as though you have a part to play in the promotion of physical activity in your Trust?
- How confident do you feel in your ability to encourage a culture of physical activity in your Trust?

**Section 3 – Set-up**

- How are new services usually embedded within the trust?
- Are there examples of multidisciplinary teams operating within the Trust?
- Who should be part of an SEM MDT team?
- What skills / qualities / previous experience are important?

**Section 4 – Implementation**

- Tell me about the SEM pilot here in Oxford…
- What has been your role within the pilot?
- Please can you describe your experience of setting up and leading this pilot project?
- How was the pilot developed?
- Who was involved in the development of the pilot?
- What changes were made within the Trust as part of the pilot?
  - How was the pilot received by…Other HCPs, Patients, Commissioners
- Who was the MDT SEM team made up of?
- How did the SEM team interact with other health professional, such as physiotherapists?

**Section 5 – Process and impact**

- What do you think worked well?
- What changes did you notice in patients?
- What changes did you notice if any, in clinical outcomes?
- What differences did you notice in the healthcare professionals involved in the different pathways?
- If you were to do this again, would you do anything differently?

**Section 6 – Barriers and facilitators**

- What or who made it easier to implement this broad programme of physical activity within the Trust?
- Who were the key players involved in getting it up and running?
- What were the barriers?
- Who else do you think could / should have been involved?

**Section 7 – Sustainability and transferability**

- How sustainable are the changes that have been made within the Trust as part of this pilot?
- What problems do you foresee for your Trust in continuing this broad programme of physical activity?
- I am a newly qualified SEM in a Trust, how would I go about implementing an SEM service like the one in Oxford Trust? Who needs to be on board?
- How important were you for the success of this pilot?
  - Would this type of thing be possible in another Trust without an SEM that is your equivalent?
- How transferrable is this model?
- Would you recommend this type of approach?
- What advice might you give to another Trust starting this process?
- Please tell me about what’s next…

**Section 8 – Close**

- Is there anything else that you have not had chance to discuss that you would like to tell me about?
- Do you have any questions for me?
